# Using network analysis for the prediction of treatment dropout in patients with mood and anxiety disorders: A methodological proof-of-concept study

**DOI:** 10.1038/s41598-018-25953-0

**Published:** 2018-05-18

**Authors:** Wolfgang Lutz, Brian Schwartz, Stefan G. Hofmann, Aaron J. Fisher, Kristin Husen, Julian A. Rubel

**Affiliations:** 1 Department of Psychology, University of Trier, Trier, Germany; 2 Department of Psychological and Brain Sciences, Boston University, Boston, MA United States; 3 Department of Psychology, University of California, Berkeley, CA United States

## Abstract

There are large health, societal, and economic costs associated with attrition from psychological services. The recently emerged, innovative statistical tool of complex network analysis was used in the present proof-of-concept study to improve the prediction of attrition. Fifty-eight patients undergoing psychological treatment for mood or anxiety disorders were assessed using Ecological Momentary Assessments four times a day for two weeks before treatment (3,248 measurements). Multilevel vector autoregressive models were employed to compute dynamic symptom networks. Intake variables and network parameters (centrality measures) were used as predictors for dropout using machine-learning algorithms. Networks for patients differed significantly between completers and dropouts. Among intake variables, initial impairment and sex predicted dropout explaining 6% of the variance. The network analysis identified four additional predictors: Expected force of being excited, outstrength of experiencing social support, betweenness of feeling nervous, and instrength of being active. The final model with the two intake and four network variables explained 32% of variance in dropout and identified 47 out of 58 patients correctly. The findings indicate that patients’ dynamic network structures may improve the prediction of dropout. When implemented in routine care, such prediction models could identify patients at risk for attrition and inform personalized treatment recommendations.

## Introduction

Premature treatment discontinuation (dropout) is a common problem in psychological interventions. There are large health, societal, and economic costs associated with high attrition rates from psychological services, particularly in the initial few sessions. From the patient’s perspective, it is associated with poor treatment outcomes^1^ and a higher hospitalization rate^2^. From a societal perspective, dropout leads to an inefficient use of clinical personnel, and strains the health system on several levels^3^. Given its economic and clinical importance, it is surprising that relatively little research has focused on dropout from psychological interventions^4–7^.

To prevent dropout and its negative effects, it is important to identify patients at risk for dropout early in treatment. Several studies have examined potential predictors of patients’ likelihood to drop out of treatment prematurely. A meta-analysis showed that dropout was significantly associated with age and education level^6^. Younger patients and patients with a lower education had an increased risk to drop out. Further findings showed that dropout was influenced by the presence of a personality disorder^2,8^, low initial global functioning, and high initial distress^2,9^. Besides patient factors, differences between therapists as well as their ability to be emotionally supportive seem to impact the probability of dropout^4,6,10^. The therapist variable, if known for a new sample, can be an additional predictor of dropout if the individual therapist effects can be reliably estimated based on enough patients per therapist in already conducted treatments. This is rarely the case in a prediction task at the onset of treatment, because in those cases the task is to predict outcome for future therapist-patient dyads and therapist effects for those new therapists are unknown. In a recent study, predictors for dropout were examined among 707 outpatients^4^. The results showed that dropout was associated with initial symptom impairment, being male, a lower educational level, negative treatment expectations, and personality style. While a more histrionic personality style increased the dropout likelihood, a more compulsive one decreased it. Consistent with this literature, we examined whether sex, education level, treatment expectancy, initial impairment, and personality dysfunction are predictors of treatment dropout.

Recently, a promising new theoretical and statistical approach to psychopathology – the *complex network* approach – has emerged with an alternative view on the structure of mental disorders^11–13^. Instead of assuming the existence of latent diseases entities giving rise to observable symptoms, the complex network approach assumes that psychological problems form complex and overlapping causal networks^14^. In a network, symptoms (nodes) are connected by their associations, which are determined by their correlations or regression coefficients. Based on these associations, the importance of individual nodes in a network is expressed via network parameters^11,15^. These parameters include measures of centrality. A highly central node is one that is likely to spread activation throughout the network via the edges connecting it to other nodes. More specifically, a node high on *degree centrality* is one with many edges connected to it; a node high on *strength centrality* is one having many edges connected to it that are great in terms of their magnitude of associations; and a node high on *betweenness centrality* is one that often lies on the shortest pathway between two other nodes. Recent research suggests that network parameters are predictive of recovery from major depressive disorder^16^.

Several studies have extended the complex network approach to intensive longitudinal assessments^11,15,17,18^. Using time series analysis techniques that consider the nested structure of the data (time points nested within patients), a network can be constructed in which nodes are connected by their time lagged associations. In a study on 53 patients with a major depressive disorder and 53 healthy controls, a higher density of their overall as well as their negative affect network over time was found for the clinical sample^19^. Furthermore, this approach enables person-by-person modeling of contemporaneous and time-lagged networks depicting idiographic topology and temporal dynamics of patient symptoms^20^.

For the computation of dynamical networks, longitudinal and high-frequency data are usually achieved via Ecological Momentary Assessment (EMA)^20,21^. EMA is a real-time and real-life within-subjects assessment of patients’ behaviors and experiences in their daily lives. Data related to the participants’ current experiences are collected a number of times a day using a mobile device, such as an electronic diary or a smartphone. The advantages over more infrequent and retrospective measurements are greater ecological validity, increased accuracy, and high rates of compliance at the scheduled time of measurement^22^, while being convenient, face-valid, and unobtrusive for the participant^23^. Daily affect dynamics measured before treatment onset with EMA were shown to predict treatment response independent of patients’ initial impairment^24^.

This proof-of-concept study aims to adopt network analysis techniques to predict dropout by individuals’ complex networks based on EMA data. More specifically, the following hypotheses guided this approach. First, we expected differentially organized group networks for dropouts and completers. This hypothesis was based on findings that symptom networks differ between patients who recover from depression and patients who do not recover during treatment as well as between clinically impaired and healthy individuals^16,19^. Second, we hypothesized that network parameters improve the prediction of dropout in comparison to patient intake variables alone. Intake variables are based on patient information at one measurement point only, whereas longitudinal networks are defined by descriptions of symptoms over time. This might allow for a better conceptualization of the symptomatology, resulting in a better prediction of dropout. Moreover, the increased utility is not merely a function of longitudinal measurements. Whereas measurements that are more traditional typically focus on level, time series network analysis recovers dynamic relationships between variables, i.e. the underlying organization of how they relate to each other in time. Therefore, patient intake variables, which have been shown to be predictive of dropout^4^, have been extended by predictors of centrality derived from network analysis.

## Methods

### Study design and participants

The sample consisted of patients who had registered for psychological treatment between October 2013 and April 2015 in an outpatient clinic in Southwest Germany. The treatments consisted of cognitive and/ or behavioral therapies. Screening for eligibility was carried out via the Mini-International Neuropsychiatric Interview 5.0.0 (M.I.N.I.)^25^ by trained clinicians via telephone. Patients were included if the screen indicated the presence of a mood or anxiety disorder based on the M.I.N.I. Of the 100 patients who were screened, 63 were eligible for study participation and 61 agreed to participate in the study. Exclusion criteria included suicidality, current symptoms of PTSD, psychosis and mania.

Eligible patients who agreed to participate were scheduled for an appointment with the study manager to sign the informed consent and to receive training for the use of the iPod for the Ecological Momentary Assessments (EMA). Patients were prompted four times a day for two weeks, every four hours between 8.00 am and 8.00 pm on weekdays and between 10.00 am and 10.00 pm on weekends, which resulted in 56 measurements per patient. After this two-week period, participants returned the iPods and received 80 € (about 89 $) as an incentive. The study was submitted to and approved by the ethics committee of the University of Trier. Since it is a non-invasive procedure, patients were informed about data protection law consequences and their opportunity to refuse to accept the storage and use of the data for research purposes at any time. Written informed consent was obtained. All methods were performed in accordance with the relevant guidelines and regulations.

After the EMA phase, the actual diagnoses were assessed after treatment onset with the Structured Clinical Interview for DSM-IV-TR Axis I Disorders (SCID-I)^26^. Interviews were conducted by trained independent clinicians with at least one year of clinical experience. These interviews were videotaped and diagnoses were discussed in expert consensus teams that included four senior clinicians; final diagnoses were determined by consensual agreement of at least 75% of the team members. During the course of the study, one patient discontinued the study, and the data of two more patients was lost due to technical problems. Data from 58 patients (3,248 measurements) were available for the analyses. After the EMA phase, treatments were part of the outpatient clinic’s routine care and patients were randomly assigned to therapists, along with all other patients who were not part of the study. Therefore, there were 35 therapists treating 1–3 study patients. All therapists in this project participated in a 3-year (full-time) or 5-year (part-time) postgraduate training program and had at least one year of clinical training before participating in the study.

### Measures

#### Dropout

Dropout was assessed routinely using the therapist’s evaluation questionnaire at the end of the treatment. An ending was considered as dropout if the patient discontinued treatment against the recommendation of the therapist before session 15. Patients were considered completers if they underwent at least 15 sessions of treatment. Dropouts had an average of 6.43 sessions (*Mdn* = 5.5, Mode = 3), completers an average of 40.31 sessions (*Mdn* = 39.0, Mode = 31). There was only one exception, in which the therapist and patient agreed to end treatment after 6 sessions.

#### Initial impairment

Initial impairment was assessed using the Global Severity Index (GSI) of the Brief Symptom Inventory (BSI)^27^, which is a short version of the Symptom Checklist 90–Revised with 53 items, and has an internal consistency of α = 0.94. The self-rated five-point Likert scale ranges from 0 (‘not at all’) to 4 (‘extremely’).

#### Personality style

The personality style was assessed using a 54-item version of the Personality Style and Disorder Inventory (PSSI-K)^4^, which consists of 14 dimensions. Although this measure is meant to assess non-pathological personality styles, high values suggest the presence of a personality disorder. The self-rated scale ranges from 0 (‘not at all’) to 3 (‘totally agree’). We used the two dimensions *careful – compulsive* and *amiable –histrionic*. They showed both internal consistencies of α = 0.71 respectively.

#### Treatment expectations

Treatment expectation was assessed with the following question: ‘How difficult is it going to be for you to be in psychotherapy?’ This question has been successfully applied in several intervention studies to predict outcome as well as dropout^4,28,29^. The answers were rated on a six-point Likert scale ranging from 1 (‘it will be very easy for me’) to 6 (‘it will be impossible’).

#### Socio-demographic variables

Patients were asked for their sex and their educational level. Sex was coded with 0 for male and 1 for female patients. Education was summarized to three categories, comprising low, middle, and high educational level. A middle educational level corresponds to junior high school, a high educational level to a general qualification for university entrance. The variable was dummy-coded with low educational level as reference category.

#### EMA variables

At each prompt, patients completed a 16-item questionnaire. Patients rated eight momentary affective states guided by the PANAS scales^30^. Patients were given the statement ‘At the moment I feel…’ with the eight adjectives *awake*, *excited*, *ashamed*, *anxious*, *depressed*, *determined*, *nervous*, and *active*, which were rated on a 5-point Likert scale ranging from 1 (‘a little or not at all’) to 5 (‘very’). Additionally, patients were asked to assess their level of rumination, worry, self-efficacy, and perceived social support in the last four hours on a ten-point scale ranging from 0 (‘never’) to 9 (‘very often’) (for items see supplementary material).

### Statistical analyses

#### Network models

All analyses were run in R 3.4.0^31^. First, three different networks based on the twelve EMA variables were modeled: the first overall network used *N*_*data*_ = 3,248 data points of all *n*_*pat*_ = 58 patients, the second used *N*_*data1*_ = 1,960 data points of the *n*_*pat1*_ = 35 completers, and the third used *N*_*data2*_ = 1,288 data points of the *n*_*pat2*_ = 23 dropouts. The networks were estimated using multilevel vector autoregressive (mlVAR) models, which regress a variable at time point t on a lagged version of the same variable and lagged versions of all other variables in the system at time point t − 1. Due to the combination with a multilevel model, the coefficients are allowed to vary between the individuals, so time dynamics can be modeled at group level and within an individual^15^. For comparability reasons, the twelve EMA variables were z-standardized and person-mean-centered before estimating the networks. For the estimation of the mlVAR models, the R package mlVAR 0.3.2 was used (for an example see supplementary material)^32^. Differences between the two networks in the 144 regression weights were tested with two-tailed t-tests for independent samples with a very conservative (*p* ≤ 0.001) and Bonferroni corrected significance level of α_corrected_ = 0.001/144 = 6.944*10^−6^. Details on the decision for Bonferroni corrected t-tests as well as on the stationarity of the time-series and the specific order as assumptions of mlVAR models are presented in the supplementary material.

#### Centrality Measures

In the next step, measures of centrality were extracted from the overall network (*n*_*pat*_ = 58 with *N*_*data*_ = 3,248), which are able to characterize the importance of specific relationships and nodes of a network. These parameters included *betweenness*, *closeness*, *instrength*, *outstrength*, and *expected force* (ExF) of each variable in each individual network, and overall density of each network^33,34^.

Betweenness relies on the concept of geodesics, which is the shortest pathway connecting two nodes in a network. The number of these geodesics (i.e. paths) between any two nodes passing the concerned one is called the betweenness of this variable. The inverse of the sum of all distances between the concerned variable and all other nodes is called closeness of this variable. The strength refers to the sum of the absolute weights of all direct connections to the concerned variable^34^. Since we modeled a directed network, strength is differentiated into instrength and outstrength: instrength is the sum of the weights of all incoming paths, while outstrength is the sum of the weights of all outgoing paths. The overall density of each network was calculated as the mean of the absolute weights in the whole network. In addition, we computed the expected force (ExF) of each variable in an individual network^33^. This metric measures the spreading power of nodes in a network by summarizing the size, density, and diversity of a node’s neighborhood in the network, while at the same time accounting for edge weight and direction (see supplementary material for more details). The centrality measures were computed using the R package qgraph 1.3.4 and the weighted ExF function^33,35^.

#### Predictor selection

As there were less than 2% missing values in the set of the seven intake predictor variables (GSI, sex, two dummy-coded variables for education level, histrionic personality style, compulsive personality style, and difficulty to attend treatment), nonparametric missing value imputation was performed (missForest 1.4)^36^. Furthermore, all raw scores of the network parameters were divided by the person mean of the respective parameter as a normalization. The normalized parameters were z-standardized to facilitate interpretability of the resulting regression weights.

To identify the most promising predictors and to increase the likelihood of reproducibility in other data, in a first step, the seven intake variables were set as predictors of dropout in a bootstrap ranking procedure with a tenfold cross-validated LASSO (least absolute shrinkage and selection operator) penalty in 150 bootstrapped samples^37^. The LASSO logistic regression model returns robust estimations of coefficients and takes correlations between the predictors into account by retaining only slightly correlated variables. We used a conservative approach with 150 bootstraps to obtain a small-sized set of the most important predictors to prevent an oversized predictors/cases ratio.

In a second step, a pool of network predictors was built out of the five network variables betweenness, closeness, instrength, outstrength, and ExF per item (5 × 12 variables), and the overall network density, resulting in 61 variables. To handle this bulk of predictor variables, we implemented machine-learning algorithms established to deal with datasets with more variables than cases. The importance of variables in the prediction of dropout was evaluated with Breiman’s random forest algorithm for classification and regression^38^. The selected variables were the first ten with the greatest mean decrease in node impurity. In line with the above mentioned procedure for intake variables, the ten extant network variables were set as predictors of dropout in a bootstrap ranking procedure with a tenfold cross-validated LASSO penalty in 150 bootstrapped samples.

To calculate incremental variance explanation in dropout, resulting predictor variables of both LASSO models were entered hierarchically into a logistic regression model (block 1: intake variables; block 2: network variables). McFadden’s *R*² for logistic regression was computed as a pseudo-*R*² estimating explained variance, and change in pseudo-*R*² was calculated to depict the supplementary usefulness of the network parameters over and above the intake variables. McFadden’s *R*² represents the ratio of the logarithmized likelihood functions of the model with explaining variables and the null model. To illustrate the predictive power of the model, the number of true/ false positive/ negative predictions was depicted using a confusion matrix as well as a receiver operating characteristic (ROC) curve. For comprehensibility reasons, the area under the curve (AUC) was transformed into more longstanding measures of effect size, using transformation rules provided by Rice and Harris^39^.

### Data Availability

The datasets generated and analyzed during the current study are available from the corresponding author on reasonable request.

## Results

### Characteristics of the study sample

Data of 58 patients was analyzed. Thirty-six patients were female (62.1%) and their age ranged from 19 to 60 years (*M* = 35.7, *SD* = 11.4). For the 58 patients, primary diagnoses as per SCID-I included affective disorders (*n* = 17; 29.3%), stress and adjustment disorders (*n* = 11; 19.0%), anxiety disorders (*n* = 5; 8.6%), eating disorders (*n* = 3; 5.2%), and other disorders (*n* = 11; 19.0%). Eleven patients (19.0%) had no diagnosis, because they dropped out of treatment before the SCID-I was performed. This is a representative sample for our outpatient clinic. Of the 58 patients, nine did not start treatment and 14 discontinued treatment before session 15. These 23 patients were considered dropouts. The remaining 35 patients were considered completers. The time between EMA and treatment onset comprised on average about 11.91 weeks (*SD* = 12.04).

### Network models

**Figure 1:**
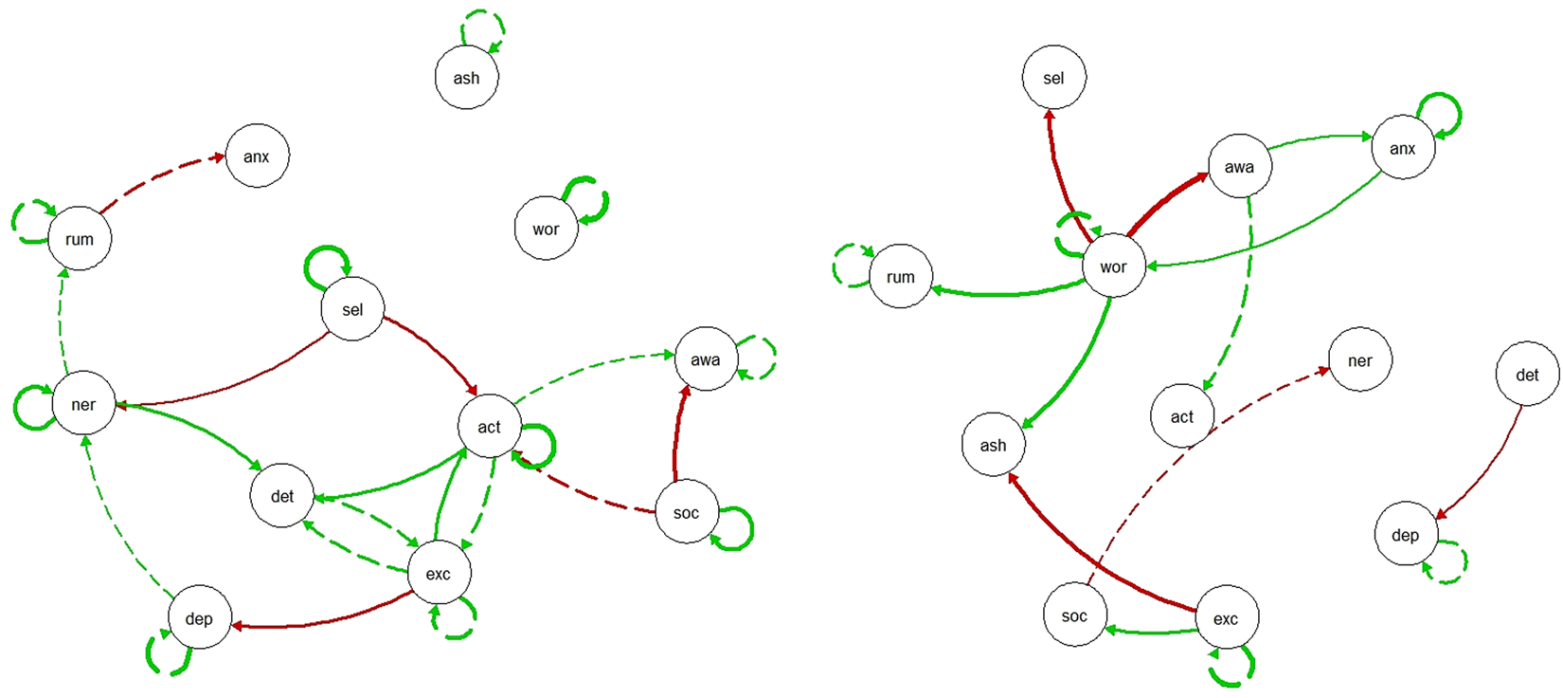
Population networks of completers (left panel) and dropouts (right panel). Green edges refer to positive and red edges to negative connections. Only edges that surpass the significance threshold (*p* ≤ 0.05) are shown. Solid edges refer to connections that differ significantly between the two networks (*p* ≤ 0.001, with Bonferroni correction), dashed edges do not differ significantly between the two networks. act = active; ash = ashamed; anx = anxious; awa = awake; dep = depressed; det = determined; exc = excited; ner = nervous; rum = rumination; sel = self-afficacy; soc = social support; wor = worry.

The networks in Fig. 1 represent the average patterns for treatment completers (network on the left) and treatment dropouts (network on the right). For clarity reasons, only significant edges (*p* ≤ 0.05) are shown. Solid edges differ significantly (*p* ≤ 0.001, with Bonferroni correction) between the networks. The completer and dropout networks differed from each other in quantity and quality. Quantitatively, there were less significant edges for the subgroup of dropouts, but the mean network density for dropouts was not significantly lower than that for completers (*M*_*completers*_ = 0.075, *M*_*dropouts*_ = 0.080, *t* = −1.11, *p* = 0.273).

Qualitatively, the two networks showed different significant edges between the nodes and most of the significant edges were also significantly different between the networks (Fig. 1). Completers who were more excited at t − 1 were less depressed and more active at t, while dropouts who were more excited at t − 1 were less ashamed and perceived more social support at t. Completers with higher perceived social support at t − 1 were less awake, but perceived even more social support at t (positive self-loop). Dropouts who worried more at t − 1 were more ashamed, ruminated more, were less awake and perceived less self-efficacy at t; associations that were not significant among completers. In contrast to dropouts, completers with an increased self-efficacy at t − 1 were less nervous and active, and reported an even higher level of self-efficacy at t. While being active at t − 1 was not significantly associated with other nodes at t among dropouts, completers who felt more active at t − 1 were more determined and active at t. Among dropouts, depressed patients at t felt less determined at t − 1, a link that was not evident for completers. Dropouts who were more anxious at t − 1 worried more and were more anxious at t; however, dropouts who were more anxious at t were more awake at t − 1. Among completers, being anxious showed no associations that differed to those among dropouts. Finally, completers who were more nervous at t felt more determined and nervous at t − 1, while dropouts showed no associations with nervous that were not present among completers.

### Dropout prediction

From the seven intake predictors entered into the LASSO model, two of them were identified as significant, namely sex of the patient (*b* = −0.32) and GSI of BSI (*b* = 0.43). When entered into the random forest model, the 61 network predictors were brought into a ranking by their importance score. In sum, the predictors with the ten highest importance scores were betweenness and outstrength of the variable nervous, betweenness and outstrength of perceived social support, expected force of awake, instrength of ashamed, instrength of active as well as closeness, outstrength, and expected force of the variable excited (supplementary material). These ten predictors were entered into a LASSO model, which displayed all significant predictors. There were four significant predictors, namely nervous – betweenness (*b* = −0.74), excited – expected force (*b* = −0.62), active – instrength (*b* = −0.68), and social support – outstrength (*b* = −0.87). The weights of all other predictors were set to zero.

We performed a hierarchical logistic regression for the two intake and the four network predictors out of the two LASSO models. In the first block, the two intake variables reached a pseudo-*R*² of *R*²_McFadden_ = 0.06, explaining 6% of the variance. Thirty-six patients (62%) were classified correctly (30 true negative and 6 true positive for dropout) and 22 patients (38%) were classified falsely (17 false negative and 5 false positive). In the second block, nervous – betweenness (*b* = −1.00, *p* = 0.018), excited – expected force (*b* = −0.90, *p* = 0.035), active – instrength (*b* = −1.02, *p* = 0.035), and social support – outstrength (*b* = −1.00, *p* = 0.029) were found to be significant predictors of dropout, whereas the two intake predictors did not reach significance level (see Table 1 for weights and *p*-values). The overall model with all six predictors had a pseudo-*R*² of *R*²_McFadden_ = 0.32. The change in *R*² from block 1 to block 2 was Δ*R*² = 0.26. The model was able to correctly identify 47 patients (81%; 30 true negative and 17 true positive for dropout), 11 patients (19%) were classified falsely (6 false negative and 5 false positive; Table 2). The Receiver Operating Characteristic (ROC) Curve showed an area under the curve (AUC) of *AUC* = 0.85 (supplementary material). This AUC corresponds to an effect size of Cohen’s *d* = 1.46 and to a point-biserial correlation of *r*_*pb*_ = 0.59. In addition, we examined a simpler baseline only network model with regard to its ability to predict a similar result as our more complex dynamic network model (see supplementary material)^40^.

**Table 1:** Fixed effects of LASSO models and hierarchical logistic regression predicting dropout.

variable	LASSO		glm			
	intake var	network var	block 1		block 2	
	estimate	estimate	estimate	*p*-value	estimate	*p*-value
sex	−0.32		−0.79	0.195	−1.27	0.101
GSI	0.43		0.87^†^	0.066	0.62	0.324
nervous −betweenness		−0.74			−1.00*	0.018
excited-expected force		−0.62			−0.90*	0.035
active-instrength		−0.68			−1.02*	0.035
social support −outstrength		−0.87			−1.00*	0.029
Δ *R*²_McFadden_					0.26***	<0.001
*R*²_McFadden_			0.06^†^	0.097	0.32***	<0.001

*Note*. LASSO = least absolute shrinkage and selection operator; glm = generalized linear model; var. = variables; GSI = Global Severity Index; ^†^*p* ≤ 0.10; *p ≤ 0.05; **p ≤ 0.001.

**Table 2:** Confusion matrix for the final model predicting dropout.

	observed	
predicted	0	1
0	30	6
1	5	17

*Note*. 0 = completer, 1 = dropout.

## Discussion

The purpose of this study was to provide a proof-of-concept for the implementation of dynamic network models to improve the prediction of dropout in psychological treatments. Predictors of dropout were examined among intake variables and network parameters. Utilizing random forest and LASSO methods, four network parameters were identified that predicted dropout better than intake variables.

Concerning Hypothesis 1, we found significantly different networks for dropouts and completers. Significant associations between the variables over time were more frequent in the completer network than in the network of dropouts. In previous studies, denser networks were associated with the existence and persistence of depressive disorders^16,19^. The authors concluded that networks with more frequent associations are more vulnerable to oscillations, which push them into a pathological state, because a worsening in one symptom causes deteriorations on many other symptoms. At the same time, it is possible that more densely connected networks can also lead more easily and more quickly to symptom improvement and even recovery if some central nodes are activated, resulting in an improvement of many associated symptoms.

Concerning Hypothesis 2, we found that network parameters could predict dropout significantly better than intake variables. The predictor selection procedure yielded a final model with two intake variables (block 1) and four network parameters as additional predictors (block 2). The two intake variables sex and initial impairment were not significant in the final model. A lower expected force of being excited, a lower outstrength of perceived social support, a lower betweenness of feeling nervous, and a lower instrength of being active were associated with a higher dropout probability.

The likelihood of dropping out of treatment was estimated as higher, if patient’s excitement influenced the symptom network less. That is, a symptom network, in which feeling excited has a larger impact on other variables in the network, seems to have protective qualities. When getting excited through treatment, these patients get more active and thus awake as well as less depressed and thus less nervous. These positive spreading processes may prevent patients from ending treatment prematurely. Furthermore, dropout was estimated as more likely, if perceived social support has less impact on other symptoms. Being embedded in close relationships and feeling socially connected has repeatedly been shown to be associated with positive health outcomes and decreased risk for premature mortality^41,42^. Social support seems to be an important external condition for improvement. Patients may tend to discontinue treatment, if perceived social support – in contrast to the health-relevant function of social relationships – does not influence their symptoms. Dropout probability was estimated to be higher when being nervous was less frequently on the shortest paths between two other symptoms. For dropouts, activation in the network containing this node does not continue its spreading process. Networks of dropouts may inhibit the spreading of change processes based on the feeling of nervousness, and thereby frustrate patients’ change expectations. Finally, dropout probability was higher when being active was influenced less by other symptoms. It may be a risk factor that there are fewer options to alter patients’ activity level, which is an important component of the treatment of mood and anxiety disorders^43^.

The final model with six predictors explained 32% of the variance in dropout probability, whereas block 1 with only intake variables explained 6% of the variance. Thus, the four network parameters improved the prediction by 26% explained variance in dropout probability, additionally to the initial impairment and the patients’ sex. A recent study on dropout in outpatients used nine predictors in the final model and was able to explain 10.55% of the dropout variance by fixed effects. Adding random effects for the therapists increased explanation of variance to 22.71%^4^. Compared to these findings, 32% of variance explained by two intake predictors and four network parameters may be classified as a large effect. Besides the status of the study as a proof of concept, the practical significance of this prediction becomes apparent when looking at the confusion matrix: Forty-seven patients were classified correctly, while only 11 patients were classified falsely. The final model was able to correctly identify 11 patients (19%) more than the block with only intake predictors.

To overcome the problem of predictor selection, we used Breiman’s random forest algorithm and a bootstrap ranking procedure with a tenfold cross-validated LASSO. Combining these two algorithms sequentially, we were able to identify four network parameters out of 61 variables. When entered into a logistic regression model, all four variables were significant predictors of dropout. The findings are initial evidence for the eligibility of these methods in psychological studies in cases of small sample sizes. In a new research situation, where a large set of variables is available, but only a subset of them includes predictive information, cross-validated LASSO models with bootstrap procedures may be a good candidate to select important predictors. This particularly applies to research topics, where a lack of previous studies impedes theoretically driven selection.

The study has some limitations. First, the most important limitation is the small sample size. Therefore, a replication and validation study in larger and representative samples is necessary. In addition to the small number of patients (*n*_*pat*_ = 58), there is a high total number of potential predictor variables (*k* = 68). These so-called ‘small n large p’ situations are relatively new to psychological applications. In other fields, such as genetics, where thousands of genes must be considered potentially relevant predictors of a certain disease, it is a common scenario^44^. Therefore, we addressed this problem by implementing machine-learning algorithms, which were developed to handle large predictor sets and have been successfully applied in DNA sequencing, epidemiology, and medicine^45^. The current sample may be small, however, in the analysis of EEG data, a random forest approach was found to be applicable to data that consisted of considerably fewer subjects^46^. Another point regarding the sample size is the unequal division of patients into completers and dropouts. The number of dropouts (*n*_*2*_ = 23) was smaller than the number of completers (*n*_1_ = 35). Accordingly, the dropout network is estimated with less statistical power than the completer network. However, it is unlikely that this differential power on its own explains the differences between the two networks, because there were not only fewer but also qualitatively different associations.

Concerning the order of autoregressive models, we focused on models with a time lag of order 1 for reasons of parsimony. Future research should expand these models by higher order time lags to examine associations with longer time intervals. Given the number of variables in the study, we had to restrict patient burden by limiting the number of questionnaires at the beginning of treatment. Therefore, we assessed treatment expectation with one item only. Since there is no standardized and generally accepted measure of treatment expectation to predict treatment outcome in psychological interventions, we used this one-item measure, which was successfully applied in several studies^4,28,29^.

Furthermore, it should be noted that the collection of EMA data is associated with considerable effort for both researchers and patients. Despite the methodological advances and the wide spread use of smart phones, not all patients are willing or able to answer questions four times a day over a period of two or more weeks. Consequently, the herein identified predictors of dropout can only be used within the subgroup of patients who do participate in pre-treatment ecological momentary assessments. However, technological advances are likely to further reduce patient burden and enhance user-friendliness of the procedure for the clinician.

The findings of the present study suggest that centrality measures of dynamic symptom networks may be able to explain more variance than intake predictors alone. Future research should investigate the effects of further variables, including therapist factors, collected before treatment onset and replicate our results. Adaptive models relying on additional process factors could be updated continuously during the course of treatment^47^. Applied methods should be expanded to face the limitations and to broaden our knowledge on the predictive strength of network analyses. This line of research can lead to direct applications to personalized and patient specific predictions of dropout in daily practice. Implemented in an app or website application, and in a diagnostic phase of intensive measurements before the onset of treatment, such network information could lead to an estimate of a patient’s specific dropout probability and be highly valuable to clinicians^48^. Combined with guidelines for clinicians how to handle patients at risk for dropout such estimates may allow reduction of dropouts, and a reduction of the costs for patients and the health system connected to it.

## Electronic supplementary material

Supplementary Information
