# Supplementary material for: Using network analysis for the prediction of treatment dropout in patients with mood and anxiety disorders: A methodological proof-of-concept study
Source: Sci Rep. 2018 May 18;8:7819. doi: 10.1038/s41598-018-25953-0 (PMC5959887; doi:10.1038/s41598-018-25953-0)
Supplement: Supplementary file 1 — Supplementary Information [file 41598_2018_25953_MOESM1_ESM.pdf]

Supplementary Information

Using network analysis for the prediction of treatment dropout in patients with mood and anxiety disorders: A methodological proof-of-concept study

W. Lutz[1*], B. Schwartz[1], S. G. Hofmann[2], A. J. Fisher[3], K. Husen[1], J. A. Rubel[1]

[1] University of Trier
[2] Boston University
[3] University of California
* correspondence to: wolfgang.lutz@uni-trier.de

EMA variables: Items

1. ‘At the moment I feel awake’
2. ‘At the moment I feel excited’
3. ‘At the moment I feel ashamed’
4. ‘At the moment I feel anxious’
5. ‘At the moment I feel depressed’
6. ‘At the moment I feel determined’
7. ‘At the moment I feel nervous’
8. ‘At the moment I feel active’
9. Rumination
   - ‘During the last four hours, I had to think about a situation permanently and wished it took another course’
10. Worry
    - ‘during the last four hours, I thought about things that could go wrong’
    - ‘during the last four hours, I thought about how things that could go wrong would influence my future’
11. Self-efficacy
    - ‘during the last four hours, I could experience my own abilities and possibilities’
    - ‘during the last four hours, I followed my own interests and needs’
12. Social support
    - ‘during the last four hours, somebody offered me concrete help’
    - ‘during the last four hours, somebody showed confidence to me’
    - ‘during the last four hours, somebody encouraged me’

Rumination was assessed using a single item. The correlations between the three items measuring perceived social-support ranged from r = ·70 to r = ·77, the correlation between the two items measuring self-efficacy was r = ·62, and the two items measuring worry correlated with r = ·92. Because of their overlap with regards to content and their significant correlations, the items measuring worry, self-efficacy, and perceived social support were averaged per construct. This procedure resulted in twelve items from EMA.

Statistical analyses: Network models

Example equation of the mlVAR model for the criterion *awake*

$$awake_{pdt} = \gamma_{0pd} + \gamma_{1pd}*awake_{pd(t-1)} + \gamma_{2pd}*excited_{pd(t-1)} + \gamma_{3pd}*ashamed_{pd(t-1)} + \ldots + \gamma_{12pd}*self\text{-}efficacy_{(t-1)} + \varepsilon_{pd}$$

$$\gamma_{kpd} = \beta_k + b_{kp}$$

where $awake_{pdt}$ is the measurement for person p (p = 1, 2, …, 58) at day d (d = 1, 2, …, 14) and time t (t = 1, 2, 3, 4), and γ_{0pd} is the intercept, which is the predicted value if all predictor variables are set to their average score (note that they are standardized and centered). γ_{kpd} represents the coefficients for awake at time t on the respective predictor k (k = 1, 2,…, 12) at time t–1, and the residual ε_{pd} is the deviation of the predicted from the observed value for awake. On level 2, β_k is the fixed effect of the lagged variable k on the criterion, and b_{kp} is the random effect, i.e. the person specific deviation of this effect. Such an equation was formed for each of the twelve EMA variables as criterion.

Statistical analyses: Network comparison

For comparing the networks of dropouts and completers, we considered to use network comparison tests. The network comparison test, which is implemented in the R package 'NetworkComparisonTest' 2.0.1 works for cross-sectional data only[1]. The function mlVARcompare of the R package mlVAR 0.4 compares the model fit only[2]. However, our goal was to quantify, which edges (associations between two nodes) are significantly different for completers and dropouts. Our efforts to find the best method for this comparison resulted in using simple t-tests with Bonferroni correction comparing the mean regression weights of the multilevel vector autoregressive analyses.

Statistical analyses: Assumptions of mlVAR models

Multilevel-VAR models are a time-series analysis technique. Therefore, some assumptions of those models have to be discussed. An important assumption is the stationarity of the time-series. Mean and variance of the time-series have to stay the same over time to comply with this assumption. We used the Kwiatkowski-Phillips-Schmidt-Shin (KPSS) test for the null hypothesis that a time-series is level or trend stationary[3]. The test was conducted separately for each of the 58 patients and 12 variables per patient using the R package tseries 0.10-43. The KPSS test indicated that the majority of time-series was level (76.40%) and trend (78.27%) stationary. Concerning the order of the model, we decided to focus on lag-1 associations for reasons of parsimony[4]. Integrating time lags of a higher order is computationally demanding, and makes the transfer of the results into clinical routine more difficult.

Statistical analyses: Centrality measures

In a demonstration study, Lawyer[5] could show the predictive accuracy of Expected Force (ExF) in predicting spreading processes, outperforming established traditional centrality measures. Divergent from the original application of ExF in social networks in which transmission follows a chronological timeline, only lag-1 associations are calculated in the current approach which is typical for psychopathological networks. Example code providing an implementation of the ExF is available at https://github.com/glennlawyer/ExpectedForce.

Results: Non-dynamic network model

We tested whether a simpler non-dynamic network based on the baseline assessments of the patients did an equally well job in predicting dropout as the dynamic network model. Therefore, we computed a non-dynamic network based on the methods used by Boschloo, van Borkulo, Borsboom and Schoevers[5] using subscales of the BSI (supplementary figure 1). Then, we compared the BSI based on the mean of the scales (unweighted severity measure) to a severity measure using scales weighted on the symptom strength of the non-dynamic network at baseline (weighted severity measure). Logistic regression analyses showed that the weighted severity measure did not improve the prediction of dropout (model 1 with unweighted severity measure: $b = 0.690$, $p = .110$, $R^2_{\text{McFadden}} = .035$; model 2 with weighted severity measure: $b = 0.789$, $p = .111$, $R^2_{\text{McFadden}} = .034$; model 3 with unweighted and weighted severity measures: $b_{\text{unweighted}} = 1.041$, $p_{\text{unweighted}} = .891$, $b_{\text{weighted}} = –0.403$, $p_{\text{weighted}} = .963$, $R^2_{\text{McFadden}} = .035$). We concluded that the results of our more complex dynamic model are worth the effort of longitudinal assessments.

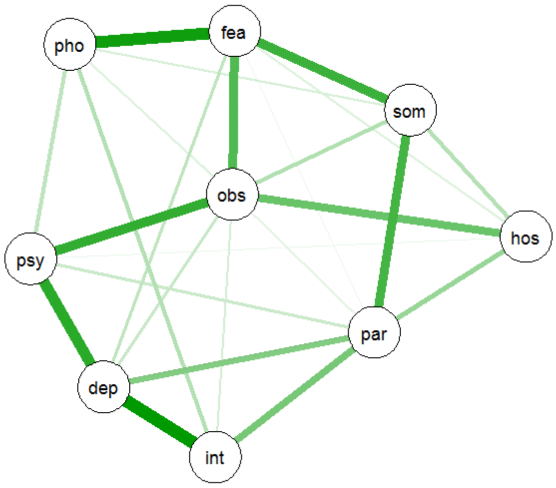

Supplementary Figure 1.Non-dynamic network model. Non-dynamic, cross-sectional network model for the subscales of the BSI. Anx = anxiety; dep = depression; hos = hostility; int = interpersonal sensitivity; par = paranoia; pho = phobia; psy = psychoticism; obs = obsessive-compulsive; som = somatization.

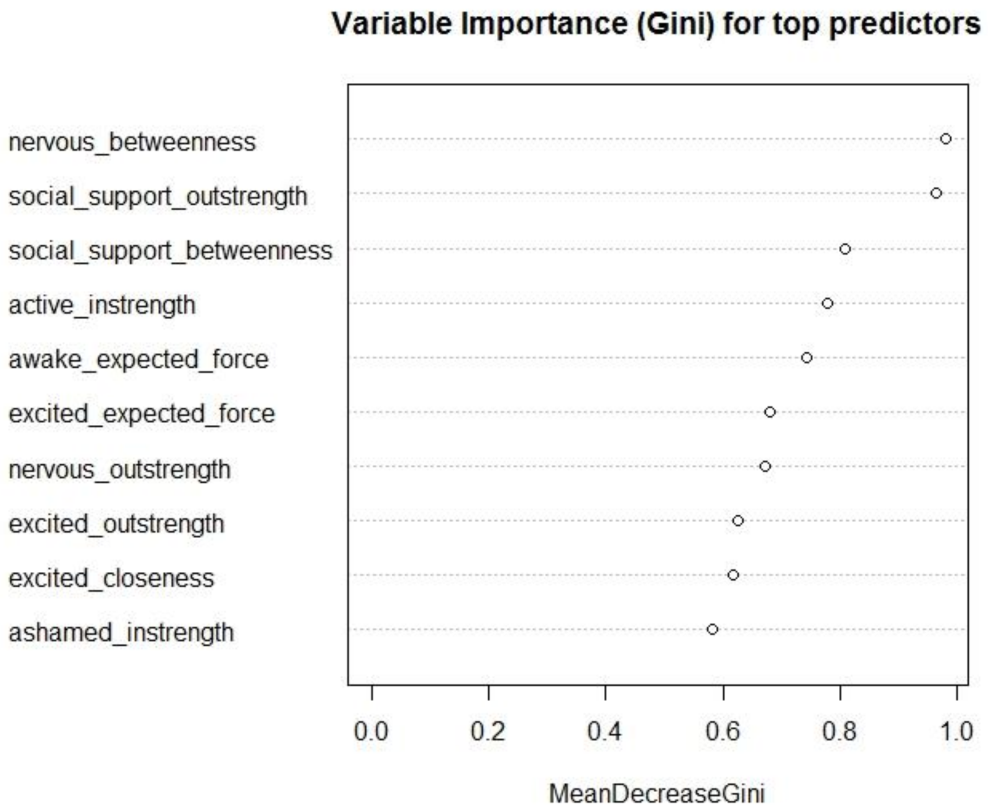

Supplementary Figure 2. **Variable importance plot.** Variable importance for the ten predictors of dropout with the greatest mean decrease in node impurity. Nervous_betweenness is the betweennness centrality of the variable being nervous, social_support_outstrength is the outstrength centrality of the perceived social support, social_support_betweenness is the betweenness centrality of the perceived social support, active_instrength is the instrength centrality of the variable being active, awake_expected_force is the expected force (ExF) of the variable being awake, excited_expected_force is the ExF of the variable being excited, nervous_outstrength is the outstrength centrality of the variable being nervous, excited_outstrength is the outstrength centrality of the variable being excited, excited_closeness is the closeness centrality of the variable being excited, and ashamed_instrength is the instrength centrality of the variable being ashamed.

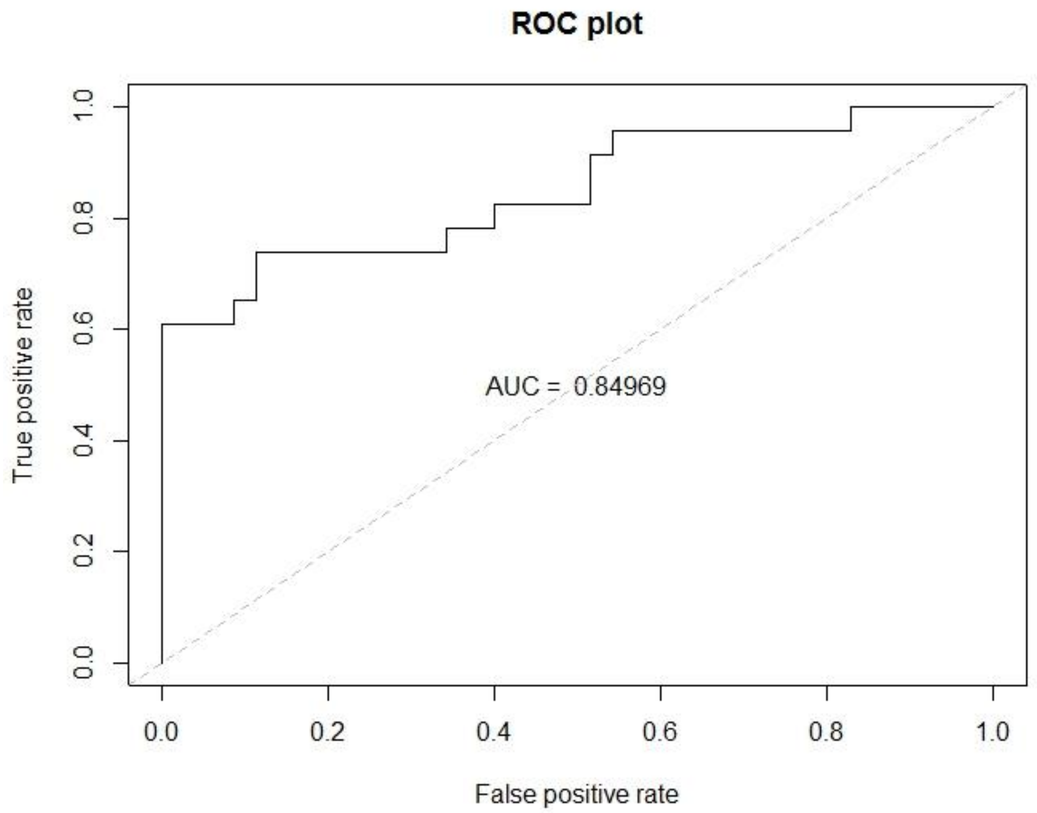

Supplementary Figure 3. **Receiver Operating Characteristics (ROC) curve.** ROC curve for the final logistic regression model predicting dropout with the false positive rate on the x-axis and the true positive rate on the y-axis.

References

1. van Borkulo, C. D. *et al*. Comparing network structures on three aspects: A permutation test. Preprint at https://www.researchgate.net/profile/Claudia_Van_Borkulo/publication/314750838_Comparing_network_structures_on_three_aspects_A_permutation_test/links/58c55ef145851538eb8af8a9/Comparing-network-structures-on-three-aspects-A-permutation-test.pdf (2016).
2. Epskamp, S., Deserno, M K. & Bringmann, L. *Package 'mlVAR'* https://cran.r-project.org/web/packages/mlVAR/mlVAR.pdf (2017).
3. Kwiatkowski, D., Phillips, P. C. B., Schmidt, P. & Shin, Y. Testing the null hypothesis of stationarity against the alternative of a unit root. *Journal of Econometrics* **54**, 159–78 (1992).
4. Bringmann, L. F. *et al.* A network approach to psychopathology: New insights into clinical longitudinal data. *PloS One* **8,** e60188 (2013).
5. Lawyer, G. Understanding the influence of all nodes in a network. *Sci. Rep.* **5,** 8665 (2015).
6. Boschloo, .L, van Borkulo, C. D., Borsboom, D., Schoevers, R. A. A Prospective study on how symptoms in a network predict the onset of depression. *Psychother Psychosom* **85**, 183–184 (2016).
